# Faculty perceptions of factors that indicate successful educational outcomes of medical students’ research projects: a focus group study

**DOI:** 10.1186/s12909-021-02954-8

**Published:** 2021-10-03

**Authors:** Riitta Möller, Annika Wallberg, Maria Shoshan

**Affiliations:** 1 Department of Medical Epidemiology and Biostatistics, Karolinska Institutet, Nobels väg 12 a, 171 77 Stockholm, Sweden; 2 Department of Oncology-Pathology, Karolinska Institutet, 171 77 Stockholm, Sweden

**Keywords:** Medical students, Scholarly research, Undergraduate research, Student thesis, Research activities

## Abstract

**Background:**

A growing number of medical schools have individual scholarly projects as a component of their curricula. The fact that all students, and not only those with research interests, have to carry out a project puts high demands on the projects and their supervision. Evidence is lacking for how to produce scholarly projects with satisfactory outcomes. This study aimed to explore the observations of faculty teachers regarding factors that predict the educational outcomes of medical students’ scholarly projects.

**Methods:**

Two focus group interviews were held with seven of the 16 faculty coordinators who were external reviewers of students’ research projects. The audio-recorded interview transcripts were analyzed using qualitative content analysis. We employed a constant comparative approach to create categories firmly grounded in the participants’ experiences. A successful project was defined as coordinators’ perception that the stated learning outcomes were achieved, in terms of students’ ability to demonstrate a scientific attitude.

**Results:**

Five categories emerged from the data: *Supervision, Project setup, Student characteristics, Curriculum structure,* and *Institutional guidance.* The supervisors’ experience and availability to students were mentioned as key factors for successful outcomes. Further, a clear aim and adaptation to the time frame were stated to be project-related factors that were also supervisors’ responsibilities. Important student-related factors were skills related to scientific writing, taking ownership of and managing the projects, and making use of feedback. Finally, the course requirements, support, and control accomplished by faculty coordinators played important roles.

**Conclusions:**

Contributing factors to achievement of the learning outcomes were supervisors’ commitment and experience, and the projects being suitable for the time frame and having a clearly stated research question. Furthermore, the students’ prowess at scientific writing, adequate handling of feedback, and ability to assume ownership of the project contributed to the final outcome, as did adherence to curricular instructions.

## Background

Medical curricula that comprise an individual research project (also referred to as a scholarly project) are increasingly common [1, 2]. In order to develop students’ skills in evidence-based medicine and medical research, they are required to carry out a research project and present a report. Some scholarly projects may be as short as a few weeks, either within the main curriculum or as an extracurricular activity [3–6]. However, a growing number of medical schools have individual projects carried out as a core curriculum component during a semester or two in real research environments [2].

Although the success of students’ research projects may be assessed as the number of publications engendered [7], a publication usually does not reflect what the individual student has actually learnt or developed. From a pedagogical point of view, it may thus be more fruitful to define success as achieved learning outcomes with regard to the usually short time frame of a student project that is expected to represent authentic and complex research. Despite the growing trend of such projects, very little is known about the factors that predict the outcome of an individual scholarly project [8].

The support a student receives during the research project is obviously of importance. While the medical school may provide lectures for all students on, for instance, statistics and research ethics, the students also receive different types and levels of individual support during their research projects [9, 10]. This type of support from supervisors (or mentors) is expected to include practical guidance, monitoring, and feedback aiming to promote the development of deeper understanding and independence [10, 11]. Universities may also have faculty members who are responsible for external review of the projects’ progression [12]. Students’ dialogues with such reviewers, supervisors, and other mentors are important checkpoints of progress and estimation of the students’ ability to attain the learning outcomes, develop research skills, present a report of reasonable scientific quality, and to finish their projects within a reasonable time. These checkpoints may also have practical implications by encouraging supervisors to improve students’ research experiences.

Sociocultural learning theories, such as communities of practice (CoP) [13, 14] and zone of proximal development (ZPD) [15], which are foundations of constructivism, constitute the framework for the present study and help to characterize the context in which research projects are carried out. These theories regard learning as participation in meaningful, authentic activities, such as interaction and collaboration with others, that develop the individuals both professionally and personally. The key underpinning concept is that ZPD requires a communicative interaction between the more knowledgeable other (e.g., a supervisor) and the learner. The supervisor’s assistance can bridge the distance between the learner’s actual developmental level and the requirements of a particular task. Self-regulation has been described as an iterative process during which learners proactively use task-specific and metacognitive strategies to plan and monitor the effectiveness of learning processes to achieve their goals [16]. Self-regulation is related to the theory of self-efficacy [17], according to which it is necessary to develop self-efficacy beliefs (beliefs that one is able to accomplish a task) to become a self-regulated lifelong learner. Thus, the heart of self-regulation —the process of setting personal goals and making adjustments to achieve the goals —depends heavily on self-efficacy beliefs [18].

There is a paucity of research on aspects influencing the outcome of medical students’ scholarly projects. A deeper understanding and characterization of successful projects may help faculty create better conditions for projects that are suitable for medical students. Thus, this study aimed to explore what faculty coordinators perceive as factors that predict the development of a scientific attitude and an understanding of scientific practice, i.e., the overall learning outcomes, in medical students’ scholarly project work.

## Methods

### Study design and setting

To explore coordinators’ experiences of monitoring students’ research projects, a qualitative approach was considered suitable [19]. The theoretical starting point was the epistemological assumption that knowledge and understanding are socially constructed [20]. The current findings were created through analysis of focus group interviews. Rather than reflecting an objective truth, the findings aim to contribute to the understanding of a complex phenomenon by providing reasonable and generalizable interpretations [20, 21].

The context of this study was a medical university with a 5.5 year undergraduate entry medical program. The first 2 years comprise basic sciences and the last 3.5 years mainly clinical education. The mandatory degree project in medicine (20 weeks; 30 European Credit Transfer System, ECTS) is placed in semester 8. All Swedish higher education is organized as sets of courses, each of approximately 1–20 weeks’ length; thus, also the medical research project is a course with its own syllabus. The overarching learning outcome is to have developed a scientific attitude, i.e., an understanding of the scientific basis of medicine and scientific practice in medical science, including the systematic acquisition and objective analysis of data, appraisal of scientific literature, and the ability to communicate and discuss the results. Therefore, the students individually carry out a research project and present, both orally and in writing, a report formatted essentially as a scientific publication. In addition to the mandatory subheadings, such as the standard sections of a research paper, the students have to write specific subsections on ethical considerations, the strengths and limitations of the study, the significance for scientific/medical/societal concepts, and aspects of equity as well as giving concrete suggestions for new research questions and future studies. The course includes about 2 weeks of initial face-to-face teaching, whereafter instruction is instead given by supervisors and their research teams. The supervisors are researchers with at least a PhD degree who can offer a project in their area of expertise. They submit a project proposal form for each student project about 6 months before the course starts, and are expected to have obtained an ethical approval for the projects before the students embark on the projects. Further, they are each expected to provide a suitable environment and sufficient resources to allow the student to carry out the project, have regular meetings with the student, and finally, act as a teacher and mentor regarding methodology, statistics, and daily activities, and to give feedback on the student’s research report during the course. Optional co-supervisors may be PhD students or other researchers in the same area.

The progress of each project is monitored by a faculty coordinator with at least a PhD degree. Each coordinator is responsible for approximately 10 students per semester and arranges three seminars (project plan, halftime, and examination) during which each student has to give an oral presentation of her/his own project and its progress. In addition, the coordinators give students criteria-based individual feedback on the projects at all seminars and thereby ensure that the projects are carried out according to the rules and regulations of our university. Coordinators also organize individual meetings with supervisors and their student(s) at the beginning of the semester in order to discuss roles and responsibilities and how much supervision the students are entitled to. As a final examination, each student presents an individual research report of about 20 pages in accordance with the university’s guidelines. Thus, the students may not present a manuscript intended for submission to a scientific journal. Two of the authors of this paper (MS, AW) have been coordinators, and all the authors (RM, AW, MS) have been course directors and examiners for the course.

### Participants

All 16 faculty coordinators of the research project course in the 2019 spring semester were invited to participate in the study. Seven of them (four females and three males, with an average experience of seven semesters as coordinators) accepted the invitation. The coordinators received oral and written information about the aim of the study stating that participation was voluntary. Informed consent was obtained from the participants at the time of the interview, and they were reassured that their anonymity would be protected.

### Data collection

Two focus group interviews were carried out by an educational developer of the medical program from March through May 2019 in undisturbed surroundings in a basic science department. Focus group interviews involve bringing together people of similar backgrounds and experiences and emphasize communication between the participants in order to generate data [22]. Semi-structured interviews with open-ended questions were used during discussions that lasted approximately 1 h. The interviewer started the discussion, moving from general to more specific questions on the coordinators’ experiences of monitoring students’ research projects. The interviews were audiotaped. The identities of the participants were anonymized prior to data analysis.

### Data analysis

The interviews were anonymized and analyzed separately. The anonymized interviews constituted the units of analysis and comprised 30 pages of text. An inductive manifest content analysis based on an iterative, constant comparison coding technique was employed to explore the collected data [23–25]. The audio-recorded interviews were transcribed verbatim, and the transcripts were checked by one of the authors (RM) and thereafter distributed to the other authors. The transcripts were then read independently by the authors to achieve familiarity with the data and to ensure accuracy.

To obtain a sense of the whole, the transcripts were read through several times and then entered into the NVivo software package [26]. Then, meaning units were identified [23]. A *meaning unit* was defined as a statement that included one piece of information of interest for the aim of the study; it could be less than a sentence but never more than five sentences. The meaning units were read through several times and each was labeled with a descriptive code. The coding was independently done by RM, but the research team members examined the transcripts in depth, and they collaboratively discussed and agreed on the initial coding. Codes that had the same or similar meaning were combined and grouped into subcategories according to their content. An overview of the process is shown in Table 1. Finally, five main categories were formulated to capture the manifest content (Table 2). Thus, codes, subcategories, and categories were developed from the text without any predetermined coding scheme. The level of the analysis was data from all the interviews rather than the views of each individual participant. Data saturation was reached, when no new categories emerged. The informants were asked to check the accuracy of the data when the analysis was completed.

**Table 1:** Overview of the steps in the analysis process

Steps in the analysis	Performed by
Transcription	RM
Review of transcription	RM
Reading of transcriptions	RM, AW, MS
Condensation to meaning units	RM, MS, AW
Group discussions to achieve consensus	RM, AW, MS
Initial coding	RM
Group discussion to achieve consensus on coding	RM, AW, MS
Initial grouping into categories	RM
Group discussion to achieve consensus on categories	RM, AW, MS

**Table 2:** Overview of the subcategories and categories generated from the interviews

Subcategories	Category
Previous experience	Supervision
Taking responsibility	
A clear research question	Project setup
Accomodation to time frame	
Ownership and management	Student characteristics
Ability to receive feedback	
Scientific writing skills	
Formal requirements	Curriculum structure
Course design	
Quality assurance	Institutional guidance
Giving feedback	

## Results

The content analysis of the interviews resulted in five main categories. These were *Supervision, Project setup, Student characteristics, Curriculum structure,* and *Institutional guidance* (Table 2)*.* All the categories are described below with illustrative quotations.

### Supervision

This category was based on two subcategories: *Previous experience* and *Taking responsibility*.

#### Previous experience

The coordinators reflected on the role of the supervisors in terms of the variation in their research experience and supervision experience. They stated that the supervisor is central to students’ projects, but that there is great variation in the amount and type of their experience. For instance, inexperienced supervisors, clinicians who defended their thesis a long time ago and are no longer active as researchers, and experienced subspecialists who plan too extensive projects more suitable for PhD students, may all be a challenge for a less-accomplished student. Thus, notwithstanding previous or current research experience, some supervisors were seen not to take into account that they were supervising undergraduate students:

*“What I reflected on a lot is the role of the supervisors, it varies so much between different supervisors; they are more or less experienced … they have more or less understanding of what it takes to supervise a medical student”. (Coordinator 1)*

#### Taking responsibility

At the initial meeting with the supervisor and the student, the coordinators outline what is expected of the supervisors, what their responsibilities are during the research project course and how much supervision the students are entitled to. One coordinator emphasized that the supervisors’ responsibility is to help the students understand the project and the methods, but that supervisors are pedagogically quite different; some focus more on students’ learning, whereas others are more interested in getting results and getting the work done. Thus, for the students, it is important what kind of supervisor they get. The coordinators also emphasized the availability of the supervisor:

*“What I expect (as a coordinator) is … that my student is in a safe context where the supervisor is available.” (Coordinator 2)*

An additional aspect brought up by the coordinators was the requirements on supervisors. Because of large student groups in the medical program, there is a high demand for supervisors, for which the only formal requirement is to have a PhD in a relevant field. At the same time, the university must guarantee supervision of good quality. Here, once again, the coordinators expressed that the great individual variation in supervision was the most difficult issue to handle and asked that the university put higher demands on supervisors’ commitment and sense of responsibility.

### Project setup

This category included two subcategories: *A clear research question* and *Accommodation to time frame*.

#### A clear research question

The coordinators reported that one indicator of a successful student project before it is even started is a clearly defined research question that can be addressed using specific, measurable entities. It was also seen as desired that the variables to be examined be clearly stated early on.

*“If the project does not have a clearly defined research question,... then it will certainly not be of good quality.” (Coordinator 3)*

#### Accommodation to time frame

The coordinators expressed that a successful student project is well-defined and well planned. It was discussed to what extent students should collect data during the course; collecting their own data is usually not difficult but takes time, which may jeopardize finishing the project withing the stipulated time. One coordinator stated that if, by the halftime seminar, students are still working only on data collection, they will usually not be able to present an acceptable final report. Thus, projects, for which data are already collected, such as register studies, were considered better suited to medical students, as they may then concentrate on other aspects of the research process. One coordinator summarized:

*“A good project is ... when you read the results, you see that it is enough … and when the project can be accomplished within the allocated time, the project should not be too limited and not too extensive; students should be able to finish the project within one semester without too many practical or other problems.” (Coordinator 4)*

### Student characteristics

This category was based on three subcategories: *Ownership and management, Ability to receive feedback*, and *Scientific writing skills*.

#### Ownership and management

The coordinators noted that students differed greatly in terms of if and how they took responsibility for and ownership of their projects, which in turn affected the outcomes of their projects. There were very determined students who had only little contact with the supervisor, and solved problems quite independently.

*“To me, this is the biggest success factor; I mean, the supervisor is important, but if you have a student who really drives the project and does everything right himself, then they hardly need a supervisor”. (Coordinator 3)*

However, some students were anxious, asked the coordinators about everything, and seemed to have difficulties working in parallel with different aspects of the research process. Others had difficulties organizing their own lives (e.g., coming to meetings on time) and had overall difficulties in achieving the learning outcomes.

Most students worked on the project every day during the semester and met their supervisors regularly, as recommended by the coordinators. This may be associated with an ability to actually assume ownership of the project. The coordinators also clarified early on to the students that they may get different feedback from their supervisor, peers, and the coordinator. Thus, the students had to understand that there are various ways to solve issues and that they have to decide what is most suitable for their project. This was described by one coordinator:

*“I make it clear to students that it may happen that they are given different advice by the supervisor, peers, and the coordinator ... so they don’t get stressed about it but understand that there are different ways to go ... that they themselves have to choose the path that seems best for the report”. (Coordinator 2)*

#### Ability to receive feedback

It appeared that students responded in different ways to the coordinators’ feedback on written and oral presentations. Some students would immediately understand and apply the feedback, while others were unwilling to make changes in their report. The coordinators had sometimes felt that their feedback had gone unheeded.

*“ With some students, you say the same thing at the beginning and at the end of the semester, the same feedback, such as you have to fix this … or keep that in mind. A whole semester in between, but the student hasn’t done anything about it”. (Coordinator 5)*

Conversely, the coordinators noted that there was also a group of very ambitious students who seemed to never have failed during their studies but who might react strongly to feedback. One coordinator emphasized the importance of a supervisor’s support in such situations.

*"They are very capable, never missed anything, and then they get some critique that they are not used to...it can be tough for them... so it’s very important that the supervisor is around and can deal with it, so the students don’t misunderstand the feedback". (Coordinator 2)*

#### Scientific writing

The coordinators agreed on that the students’ skills in scientific writing were important for the overall success of the project, but the level of skills varied. They exemplified this with an ability or inability to uphold the central theme and remain focused throughout the entire paper.

*“To write in a structured manner with a main theme is difficult … sometimes, I do not understand, not even when they write in Swedish. Then, I wonder, how do they think when they can’t express themselves logically? I do not understand … ”. (Coordinator 6)*

Scientific writing and stringency are connected. In the earlier versions of their reports, some students include quite irrelevant information, speculate and are too quick to draw conclusions. One coordinator pointed out that it is essential to make students understand that their personal reflections do not belong in scientific texts. Furthermore, it was underlined that lucid scientific writing is an indication that the student has developed a scientific attitude:

*“ We all emphasize that the reader shouldn’t have to guess … .the text should be understood by everyone in the same way … and it is only then we know that the student has adopted a scientific approach”. (Coordinator 3)*

*“The writing and the stringency are connected … you may deceive yourself that everything is clear in your head ... but you don't really know that until you have to write it down ... so writing is important as the requirements [of our course ]are quite high” (Coordinator 3)*

### The curriculum structure

This category was based on two subcategories that emerged from the interviews: *Formal requirements* and *Course design.*

#### Formal requirements

The coordinators reflected on the formal requirements for students’ research reports and noted that a limitation on the number of pages would be beneficial, as some reports were too long. In addition, overly long texts discourage students from reading one anothers’ reports in their entirety, despite the compulsory peer review they must present at the final seminar. In accordance with the instructions, new subsections, on topics such as gender equity in research and the significance of the project for medical science and/or society, do increase the length of the reports and make them less publication-like. The coordinators also perceived that the required number of pages triggers distress among students. Altogether, the coordinators preferred a shorter research report.

*“All these extra subheadings... the report should be about the research objective...and we should teach students to stick to what is relevant”. (Coordinator 3)*

#### Course design

In order to help students to attain the goals, some formal teaching is arranged during the course. The coordinators stated that tuition in basic statistical methods should be included in the course and that students must learn how to do these analyses themselves for in-depth understanding. Students also need to be taught how scientific papers are structured and to become discerning about scientific quality in papers. One coordinator recommended that such learning activities should preferably be arranged during the second half of the course, by which time the students should have collected and read a library of project-related publications. This would benefit their appreciation of the training and their participation in discussions. Furthermore, the coordinators recommended that instruction and training in scientific writing should be mandatory and that it should focus on the coherency of the report:

*“I think the teaching in scientific writing should focus on title, purpose, introduction to discussion and conclusion. If these parts do not hold together, then we can stop reading, you see”. (Coordinator 7)*

Regarding the assessment and feedback procedures of the course, the coordinators appreciated the rubrics with descriptors and described that the students also used them in peer review of other students’ reports. The use of rubrics makes the assessments more standardized and fair, and helps the coordinators to keep track of key aspects of the written reports so that they will fulfill the requirements.

*“Using criteria is beneficial, it makes the assessments more standardized, although you cannot implement them to 100 % because all projects are unique”. (Coordinator 2)*

### Institutional guidance

This category was based on two subcategories: *Quality assurance* and *Giving feedback*.

#### Quality assurance

The coordinators held that their task was to ensure the quality of the projects and the reports. Early in the course the coordinators reviewed the students’ project plans and emphasized the importance of a stringent description of the current state of knowledge in the field and the identification of a knowledge gap. They also considered it important that students understand the assumptions and conditions of the project, and would therefore ask students about, for example, the methods, variables, and hypotheses.

*“In the first seminar, when we review the project plans, we have to ask questions about the research question: Do they have a hypothesis or not? Does the study have an explorative approach? What are the specific research questions?.., here, I think our role is very important”. (Coordinator 1)*

The coordinators also stated that their task was to clarify for students and supervisors their roles and responsibilities. They also reported that they sometimes have to negotiate between the student and the supervisor, and explain that they all want the student to do well in the course.

*“Sometimes, it happens that I have to mediate a little between the supervisor and the student ... and, in these situations, it is important that there is trust, that everyone is aiming for the same goal”. (Coordinator 1)*

#### Giving feedback

The coordinators described the importance of their individual feedback to students, for instance to students with less-available supervisors, or in order to emphasize the instructions. One coordinator expressed that she tried to focus on one major message for each student for improving his/her report. Another coordinator stated that it is important to point out everything that the student could do better, not because something is necessarily bad but because they want the students to produce final reports that are as good as possible. It was also expressed that giving feedback is sometimes emotionally challenging in terms of the varying levels or types of feedback from one project and student to another.

*“ They think that I’m the difficult person … in one case, I’m trying to delimit the project, in another, I’m trying to structure the project so that it is possible to carry out”. (Coordinator 6)*

According to the coordinators, an important advantage was that they have met many students over several semesters. In this way, the factors that are important to students’ success are sometimes clearer to them than to the supervisors, especially regarding the students’ learning perspective. One coordinator described how he sees himself more as a coach whose feedback helps the students understand what is meant by good quality and what would downgrade any scientific report.

## Discussion

This study focused on factors that from an educational viewpoint indicate a successful outcome of the increasingly common curricular research projects for medical students. Rather than assessing the outcome as the number of resulting publications, which has been done in several previous studies [8, 12, 27], we report faculty teachers’ (coordinators’) perceptions of students projects that are successful in terms of developing students’ scientific attitude and skills as revealed by qualitative analysis of focus group interviews. The interviews showed that the main contributing factors were supervisors’ previous experience and availability for the student, a clear research question, well-defined variables, and accommodation to the time frame. Important student-related factors were scientific writing skills, appreciation of feedback, and developing ownership and management of the projects. Finally, adherence to curricular instructions and coordinators’ counsel also contributed to the final outcome.

That supervision was found crucial to the outcome of the students’ scholarly projects is in line with previous research [28, 29]. However, supervisors’ experience and practices vary, which may impact the quality and progression of the projects. This is reflected in the finding that the focus of the supervisors was reported to vary; some of the supervisors seemed to focus on the product (i.e., to get the research work done), while others focus more on the process (i.e., students’ learning). While variability in supervisory practice has to be accepted as part of adapting it to each student, combining these two approaches — i.e., supporting students’ learning and development of autonomy as well as producing a research according to the requirements — would be a preferred model [30].

Many academics’ first supervisory experience is with undergraduate students, and, without access to support or training, the situation may be stressful for supervisors and students alike [29]. Supervising undergraduate students may actually be more challenging than supervising doctoral students, as supervisors have to balance instruction and student independence, while keeping in mind the time constraints [30, 31]. In addition, undergraduate students usually have little previous research experience, and may encounter difficulties in transitioning from regular coursework to more independent project work and more self-regulatory learning [32], along with having variable aptitudes for the task, and focusing on a professional clinical career rather than research [29] at this stage of their education. Supervisory inexperience with these types of challenges indicates a need for faculty development programs and support for new supervisors [30].

Our results reveal that a student’s ability to lead and propel his/her project, i.e., to develop autonomy, is one indicator of a successful outcome. Indeed, every profession relies on the ability of its members to self-regulate their performance in order to achieve excellence [18, 32, 33]. As scholarly projects offer an opportunity to develop self-regulation and autonomy in learning, supervisors should be encouraged to support students in developing these processes by encouraging them to ask questions, critically appraise new information, identify their learning needs and gaps in their knowledge and, perhaps most importantly, to reflect on their own learning process and the learning outcomes [34].

In medical practice, as well as in research, clear and accurate oral and written communication is essential. This was an important learning outcome of the course, and therefore, the students were expected to produce an individually written report formatted as for peer-reviewed journals. Our interviews revealed that the quality of the report depended on the student’s ability to uphold a central theme and to achieve scientific stringency throughout the report. Pedagogical practices for the development of writing are, however, often overlooked and rarely practiced in medical education before the scholarly project course when the requirements are quite high [35, 36]. Miller et al. [37] found in their study among nursing students that writing competence improves when students are guided and given opportunities to practice. The study concluded that the challenge in teaching scholarly writing is to connect writing and critical thinking to a context. This is in agreement with the present study, suggesting that without critical analysis and a context, the writing assignments will only be exercises in grammar and syntax. These and other findings suggest that scholarly writing sessions, including feedback, should be integrated into research project courses in order to support students’ evaluation of their own writing skills [38], and that supervision should include feedback and critical editing of multiple drafts of the project report [35, 36].

The aim of feedback should be to benefit the student by reducing possible gaps between current performance and the learning outcomes [39]. One challenge pointed out by the coordinators in the present study was how students handle feedback. Adequate appreciation of sometimes contradictory feedback from coordinators and supervisors requires a self-regulated student who takes control of her/his learning and actively interprets external feedback [32, 40]. Self-regulation is related to self-efficacy, which refers to students’ beliefs and attitudes toward their capabilities to fulfill given task demands and is shaped by self-beliefs about one’s skills in a specific context [17, 32]. As part of the process of developing self-regulation skills, it is indeed essential that students learn to reflect on the learning outcomes and to make judgments about how their work relates to the criteria that will apply to their work [40]. Even if presented only in conjunction with report writing, feedback and making use of it are also aspects of ZPD [15] and CoP [13, 14], as scientific writing must reflect the objectivity, the theoretical underpinnings and the logical conclusions of a project and the community behind it. Research project courses should thus inform students how criteria will be applied to their work. Furthermore, future faculty development courses should not only focus on the feedback itself, but also address how teachers can empower students self-efficacy and and use feedback effectively.

Several limitations in our study warrant a discussion. Firstly, its credibility may be questioned, as our data were derived from focus groups and confined to individual perspectives and coordinators’ subjective descriptions [21, 23]. However, both positive and negative aspects were revealed, indicating that the coordinators felt comfortable enough to express their true opinions. Those who volunteered may not be representative of the whole group; it is possible that other coordinators might have had other valuable experiences. However, given the large number of students and the participating coordinators’ experience, we consider the results trustworthy. Investigator triangulation was used, engaging all the authors in the analysis. The coding and the definition of categories were discussed and checked by all the authors. To attain dependability, data were collected until no new categories emerged (saturation) and were continually re-examined using the insights that emerged during analysis. The researchers were closely involved in the research setting and may have brought particular assumptions to the research. Therefore, to establish confirmability, the informants were asked to check the accuracy of the data when the analysis was completed [21]. Finally, this study was conducted at a single institution, for which reason the context has been described at some length [23]. This enables the reader to evaluate transferability to other settings.

### Future studies

We focused on faculty teachers’ experience of students’ research project guidance, by using focus group interviews as a data source for qualitative analysis. Future studies should use different data sources, e.g., students and supervisors, to obtain further understanding of succesful projects. It would also be valuable to use other methods, such as questionnaires to previous students, to explore their experiences of supervision related to the stated learning outcomes of the course. Likewise, a broader investigation of supervisory practices is merited to illuminate how to support and develop students’ skills and self-regulatory learning.

## Conclusions

Contributing factors to successful development of a scientific attitude and understanding of scientific practice during a scholarly research project included supervisors’ previous experience, their availability to the student, a clearly stated research question, well-defined variables, and accommodation to the short time frame. Furthermore, students’ prowess in scientific writing, adequate handling of feedback, and ability to assume ownership of the project contributed to the outcome, as did adherence to curricular instructions. Therefore, faculty support for less-experienced supervisors could be valuable to create good conditions for students’ research projects. In addition, students should receive training in scientific writing and in assuming ownership of their projects. With the aim of integrating supervisors’ and students’ views on successful project outcomes, future studies should compare their perceptions. In order to elucidate their perceptions of learning outcomes and scientific writing, further studies could also focus on how students interpret the criteria that are used to assess their reports.

## Data Availability

The datasets generated during and analysed during the current study are not publicly available due to the fact that although the interview transcripts are anonymised, it is with some knowledge of the context not impossible to identify the participants. However, the datasets are from the corresponding author on reasonable request.

## Abbreviations

CoP: Community of Practice
ECTS: European Credit Transfer System
ZPD: Zone of Proximal Development
